# Palliative care strategies in the management of people with serious cases of COVID-19

**DOI:** 10.1590/0034-7167-2022-0308

**Published:** 2023-03-06

**Authors:** Eleandro do Prado, Camila Wohlenberg Camparoto, Angelica Yukari Takemoto, Sueli Mutsumi Tsukuda Ichisato, Maria Emília Grasso Busto Miguel, Sonia Silva Marcon

**Affiliations:** I Universidade Estadual de Maringá. Maringá, Paraná, Brazil

**Keywords:** Palliative Care, Health Strategies, Coronavirus Infections, Pandemic, COVID-19., Cuidados Paliativos, Estrategias de Salud, Infecciones por Coronavirus, Pandemia, COVID-19., Cuidados Paliativos, Estratégias de Saúde, Infecções por Coronavírus, Pandemia, COVID-19.

## Abstract

**Objectives:**

to analyze the assistance strategies in palliative care developed in the COVID-19 pandemic for critically ill patients and their families.

**Methods:**

an integrative review carried out in August 2021 and updated in April 2022 in the *Base de Dados de Enfermagem* (BDENF), Cumulative Index to Nursing and Allied Health Literature (CINAHL), Medical Literature Analysis and Retrieval System Online (MEDLINE), US National Library of Medicine (PubMed), Web of Science databases, and presented in the PRISMA flowchart.

**Results:**

thirteen works were selected for reading and content analysis, from which emerged the two main themes that reflect the reality evidenced in this context: The sudden advent of COVID-19 with impacts on palliative care; and The strategies used in palliative care to mitigate these impacts.

**Final Considerations:**

palliative care is the best strategy for providing health care, used as a comfort to bring relief and comfort to patients and families.

## RESUMO

**Objetivos:**

analisar as estratégias assistenciais em cuidados paliativos desenvolvidas na pandemia de COVID-19 aos pacientes graves e familiares.

**Métodos:**

revisão integrativa, realizada em agosto de 2021 e atualizada em abril de 2022, nas bases de dados Base de Dados de Enfermagem (BDENF), *Cumulative Index to Nursing and Allied Health Literature* (CINAHL), *Medical Literature Analysis and Retrieval System Online* (MEDLINE), *US National Library of Medicine* (PubMed), *Web of Science*, e apresentadas no fluxograma PRISMA.

**Resultados:**

foram selecionados treze trabalhos para leitura e análise de conteúdo, dos quais emergiram as duas principais temáticas que traduzem a realidade evidenciada neste contexto*: O advento súbito da COVID-19 com impactos nos cuidados paliativos*; e, *As estratégias utilizadas em cuidados paliativos para amenizar esses impactos*.

**Considerações Finais:**

os cuidados paliativos se convergem na melhor estratégia de prestação de atendimento à saúde, usados como um acalento para trazer alívio e conforto aos pacientes e às famílias.

## RESUMEN

**Objetivos:**

analizar las estrategias asistenciales en cuidados paliativos desarrolladas en la pandemia de COVID-19 para pacientes críticos y sus familias.

**Métodos:**

revisión integradora, realizada en agosto de 2021 y actualizada en abril de 2022, en las bases de datos *Base de Dados de Enfermagem* (BDENF), *Cumulative Index to Nursing and Allied Health Literature* (CINAHL), *Medical Literature Analysis and Retrieval System Online* (MEDLINE), *US National Library of Medicine* (PubMed), *Web of Science*, y presentado en el diagrama de flujo PRISMA.

**Resultados:**

se seleccionaron trece obras para lectura y análisis de contenido, de las cuales surgieron dos temas principales que reflejan la realidad evidenciada en este contexto: El advenimiento repentino de la COVID-19 con impactos en los cuidados paliativos; y Las estrategias utilizadas en cuidados paliativos para mitigar estos impactos.

**Consideraciones Finales:**

los cuidados paliativos son la mejor estrategia para brindar atención en salud, utilizados como un consuelo para brindar alivio y comodidad a los pacientes y familiares”.

## INTRODUCTION

Humanity has been facing outbreaks of diseases that plague the world population, such as the H1N1 virus pandemic, in 2009, of Ebola, in 2013, and that of SARS-CoV-2, more recently, called Coronavirus Disease 2019 (COVID-19). The latter belongs to family *Coronaviridae*, being responsible for spreading an infectious disease with highly transmissible characteristics that reached, in March 2020, epidemiological levels, to the point of being declared a pandemic by the World Health Organization (WHO)^(1)^.

Since the first records, this disease has accumulated thousands of infected, dead and cured, currently, not only the initial virus, but its numerous variants continue to worry countries and health authorities. To contain the spread of the disease, sanitary protocols were adopted, being constantly renewed as safety measures to reduce its circulation, such as social distancing, use of masks, frequent hand hygiene and availability of vaccines^(2)^.

Although the estimate that 70% to 80% of those infected are asymptomatic or present mild symptoms of the disease, around 20% progressed to more severe forms and 5% to 10% could progress to conditions that will require intensive care or will present clinical irreversibility, especially in older adults, underlying medical conditions, such as cardiovascular disease, diabetes, respiratory disease, obesity, among others, that are classified as prone to developing the most severe forms of COVID-19^(3)^.

Initially, by not having a clear clinical description or known pattern of lethality, the number of COVID-19 cases expanded in a short period of time around the world and soon turned into an unprecedented humanitarian crisis. This exponential increase in the number of infected and severe cases has put at risk the structures of care services as a result of the imbalance between supply and demand for intensive care beds^(4)^.

This scenario brought to the center of the discussions a delicate and little debated question in society, regarding the end of life, which in this case was transvested with the question that prevailed at the beginning of the pandemic, when knowledge about the disease was still incipient: to whom and until when to ensure assistance and resources for the recovery of conditions considered serious and irreversible by COVID-19^(3)^?

It is common to have care gaps like this in times of humanitarian crisis. In this sense, the fundamentals that guide palliative care take on the leading role, being ethically decisive for optimizing the indications of available resources and helping to resolve deficiencies exposed in the health area, since the planning and implementation of its actions are based on suffering assessment and mitigation^(5-6)^.

Palliative care is defined by the WHO as an approach whose objective is to improve the quality of life of patients and their families, through the prevention, relief and rapid identification of suffering, in addition to the assessment and treatment not only of physical symptoms, but also emotional, psychosocial and spiritual ones^(7)^.

Considered a fundamental strategy in the treatment of COVID-19, the WHO included an update in the form of a chapter in the document “clinical management of COVID-19” with recommendations that reinforce the need for strategies that promote access to palliative care during the pandemic. In order to assist health professionals who are in the front line, this update brings an expansion of palliative care to meet the various demands and promote a multidisciplinary approach to the emerging needs of patients with coronavirus in different degrees of commitment and their families^(7)^.

Thus, including palliative care, more than a viable option, is a necessity not only to ensure the quality of care and ethics in the resolution of cases that require firmness of decision, but to ensure the right to dignity and comfort in the face of a life-threatening disease with uncertain progress and prognoses^(8)^.

## OBJECTIVES

To analyze and synthesize scientific evidence regarding the care strategies developed during the COVID-19 pandemic by health teams in palliative care in favor of patients and their families.

## METHODS

This is an integrative literature review, which allows the synthesis, identification and more specific analysis around a specific phenomenon already described in the literature, as well as pointing out possible gaps that can, through new research, be solved^(9-10)^.

**Figure 1:**
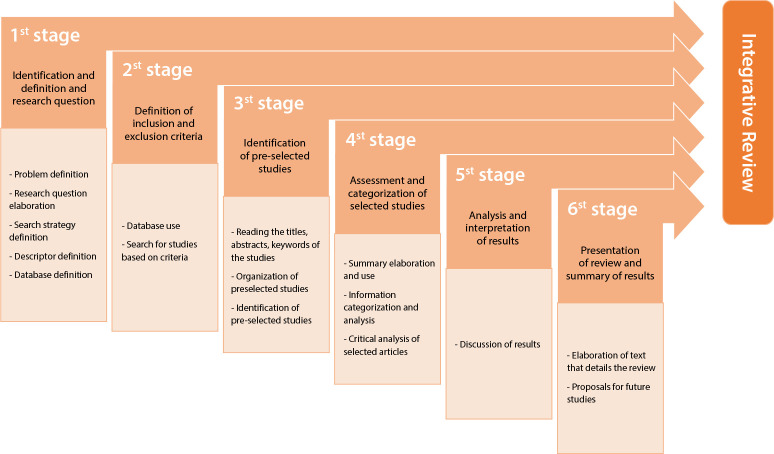
Stages for selecting articles, Maringá, Paraná, Brazil, 2022

To ensure rigor in methodological conduction, the search strategies were performed by independent peers (E.P. and K.M.), standardizing the sequence of descriptors and crossings in the databases of the Journal Portal of the Coordination for the Improvement of Higher Education Personnel (CAPES - *Coordenação de Aperfeiçoamento de Pessoal de Nível Superior*), with access through the Federated Academic Community (CAFe - *Comunidade Acadêmica Federada*), and reviewed together to define inclusion and exclusion. The construction of this research was structured in six stages, confluent to the method and as proposed methodologically (Figure 1)^(11)^.

In the first stage, the question that guided the investigation was formulated, based on the search strategy known as the acromion PICo^(12)^, where: (P) Population: people with severe clinical conditions of COVID-19; (I) Interest: palliative care strategies adopted by a multidisciplinary team; and (Co)Context: COVID-19 pandemic. In this sense, the following review question was formulated: what are the palliative care strategies used during the COVID-19 pandemic by a multidisciplinary team for people with severe cases of the disease?

Data collection was carried out in August 2021 and updated in April 2022, with an advanced search in the Cumulative Index to Nursing and Allied Health Literature (CINAHL), *Base de Dados de Enfermagem* (BDENF), Medical Literature Analysis and Retrieval System Online (MEDLINE), US National Library of Medicine (PubMed), Web of Science databases. The descriptors were delimited, according to the Medical Subject Headings (MeSH) (Palliative care AND health strategies AND Health professionals AND COVID-19) and Descriptors in Health Sciences (DeCS) (in Portuguese) (*Cuidados Paliativos* AND *Estratégias de Saúde* AND *Profissionais de Saúde* AND COVID-19). To carry out the search, the descriptors were also used specifically by “Title/Text”, integrating them through the Boolean AND operator, as shown in Chart 1.

**Chart 1:** References found at the respective intersections (1^st^ search and subsequent update) (N=144), Maringá, Paraná, Brazil, 2022

Database	Search strategy	Nº of articles
Portal Regional da BVS: (BDENF, MEDLINE)	Title/Abstract/Text: (palliative care) AND (health strategies) AND (COVID-19)	61
PubMed	All=(((palliative care) AND (health strategies)) AND (Health professionals)) AND (COVID -19)	32
Web of Science	((ALL=(palliative care)) AND ALL=(Health strategies)) AND ALL=(Health professionals)) AND ALL=( COVID-19)	20
CINAHL	TX palliative care AND TX health strategies AND TX health professionals AND TX COVID -19	31

*TX - text; COVID-19 - Coronavirus Disease 2019.*

A script was used by the researcher, which was adapted from a validated instrument^(11)^ to collect data and standardize the information abstracted from each work, grouping the syntheses and later facilitating the elaboration of categories, according to Chart 2.

**Chart 2:** Script adapted for data collection and organization^(11)^, Maringá, Paraná, Brazil, 2022

Title	Publication year	Study objective	Conclusions	Study design

Only original articles were included, published in full and that addressed the palliative care strategies that were evidenced by health professionals during the COVID-19 pandemic. The choice of languages was restricted to Portuguese, English and Spanish, and the limited time for the search took place from November 2019 to 2022, considering the beginning of the COVID-19 pandemic as a time frame. Literature was excluded, such as dissertations, monographs, theses, books, editorials, manuals, books, among other non-indexed references.

Finally, after grouping, the results were submitted to the synthesis of qualitative evidence, which was based on content analysis^(13)^, so that the scientific evidence around the palliative care strategies used by health professionals during the COVID-19 pandemic could be extracted in a succinct and systematized way. This analysis process took place through a dynamic process of coming and going from the information extracted from the manuscripts, facilitating the synthesis of the content and the presentation of this integrative review.

## RESULTS

The initial search resulted in 144 publications in the searched bases, according to the distribution: BDENF (n=03); PubMed (n=32); CINAHL (n=31); MEDLINE (n=58); Web of Science (n=20). After the search, the results were treated according to the established selection criteria, resulting in the inclusion of 13 articles analyzed in this review.

**Figure 2:**
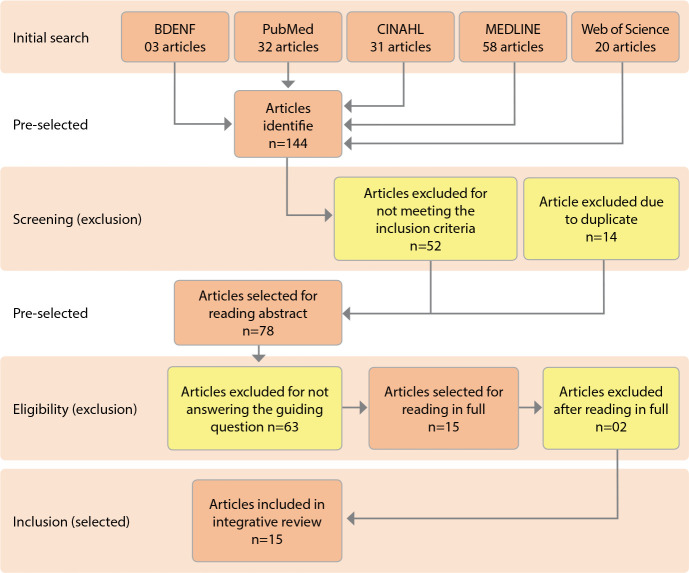
Flowchart of selection of articles in the databases adapted from (PRISMA)^(14)^, Maringá, Paraná, Brazil, 2022

To better organize and systematize this process of searching and selecting publications, the Preferred Reporting Items for Systematic Reviews and Meta-Analyses (PRISMA)^(14)^ methodology was used, whose stages are demonstrated through the flowchart, described in Figure 2.

The 13 selected articles portray the strategies used by health professionals around the world to humanize care through palliative care for patients affected by serious and irreversible clinical conditions of COVID-19. Of these works, six were published in 2020 (46%) and eight in 2021 (54%). As for the place where they were carried out, the search revealed a concentration of studies in the United States of America, which corresponds to six works (50%). The other works were carried out precisely in countries that also had high levels of contamination on the world stage, such as England (14%), Germany, India, Italy, Ghana and Canada, equally with (7%) each, and it should be noted that all articles included in the survey are international. As for the methodology, the studies showed a predominance in the exploratory-qualitative approach (57%).

To facilitate a better visualization of results, a chart was created describing article title, year of publication, objective, conclusion with the main study evidence and method used in the study (Chart 3).

**Chart 3:** Summary of article characteristics according to title, authors, year of publication, place of research, study objective of study and design, Maringá, Paraná, Brazil, 2022

Title	Year of publication	Study objective	Conclusions	Study method
Experiences, challenges and perspectives for ensuring end-of-life patient care: A national online survey with general practitioners in Germany^(15)^	2021	Describe the experiences, strategies and challenges faced by physicians in COVID-19 care.	Remote communication has become the main strategy to keep the patient close to the family even during hospital isolation. This strategy gained evidence by ensuring not only the humanization of care, but human dignity at the end of life, giving patients and family members the opportunity to be close.	Qualitative exploratory study
Creating a Palliative Care Inpatient Response Plan for COVID-19 The UW Medicine Experience^(16)^	2020	Share strategies created by health professionals in the implementation of high-quality palliative care in the context of contingency and crisis as a result of COVID-19.	The speed with which the contingency plan was instituted for the care of patients without palliative care during the height of the crisis proved to be largely effective in relieving severe symptoms, supporting family members, especially in the use of remote approaches.	Descriptive-exploratory study
Palliative Care Interventions from a Social Work Perspective and the Challenges Faced by Patients and Caregivers during COVID-19^(17)^	2020	Describe challenges faced by patients under palliative care and their caregivers during COVID-19 pandemic restrictions and what interventions are provided by the team to face these conditions.	It highlights the positive lessons experienced by a palliative care team, with an emphasis on support strategies, highlighting the importance of maintaining contact between the family and patients, even remotely, especially in times of gravity and grief.	Qualitative exploratory study
Lessons Learned from caring for patients with COVID-19 at the end of line^(18)^	2021	Describe the experiences of a multidisciplinary team in palliative care when living with the death of patients infected by COVID-19	It is concluded that to face challenges, such as the pandemic, palliative care teams must adapt quickly, be agile, innovative and open to new ideas. Care strategies such as those presented here, through remote visits and phone calls, helped to mitigate the distance between patients and their families, alleviating distance, the death and dying process and anticipated grief.	Descriptive study
“Why Couldn’t i go in to see him?” Bereaved families’ perceptions of end-of-Life communication during COVID-19^(19)^	2021	Analyze the perceptions of bereaved families about the quality of end-of-life communication between health professionals during the COVID-19 pandemic	The study shows that low-quality communication increases suffering and affects the quality of dying and mourning. The results underscore that remote communication at the end of life was an excellent strategy to maintain access to the team, families informed and close through face-to-face contact with terminally ill patients. In addition, it is also the most significant strategy given the limitations and restrictions imposed by the pandemic.	Descriptive-qualitative study
Learning a palliative care approach during the COVID-19 pandemic: A case study in an Infectious Diseases Unit^(20)^	2020	Describe the consultation and assistance intervention of a palliative care unit during the COVID-19 pandemic, determining which changes are necessary for palliative care provision.	It was identified that some elements of conventional palliative care that needed to be readapted in the pandemic scenario: Improvement in communicating bad news; optimization and use of communication devices; agility in critical interventions and guarantee of a peaceful death in the hospital.	Descriptive-qualitative study
Communication and virtual visiting for families of patients in intensive care during the COVID-19 pandemic: A UK National Survey^(21)^	2021	Understand how communication between family members, patients and ICU staff during the pandemic was made possible and explore the strategies used to facilitate virtual visit	There were changes in the way ICU teams began to communicate with patients’ families during the pandemic, some created communication teams and others adopted a virtual visit system. The therapeutic benefits of these alterations went beyond the informative and emotional support to the family, becoming an essential aid in patients’ recovery, in the team grieving process and morale. On the other hand, a barrier to be considered in the implementation of these resources was also evidenced, the lack of access or ability of many families to these virtual devices, which, instead of bringing them closer, can segregate them.	Cross-sectional study
Hearts above water: Palliative care during a pandemic^(22)^	2021	Describe the aspects involved in nursing approaches during palliative care assistance that were structured to foster humanistic patient and family care amidst social distancing and visitation restrictions in the context of the COVID-19 pandemic.	After experiencing numerous challenges during the COVID-19 pandemic and realizing the need for knowledge regarding advance directives and knowledge about the death and dying process, palliative care teams began to engage in the dissemination of preventive information, especially in relation to advance directives, and emphasize the importance of talking about the subject in families, in the community, hoping to raise awareness of the need for advanced planning that will certainly help in new crises like these.	Qualitative exploratory study
Urgent creation of a palliative care team in a small hospital during the COVID-19 crisis^(23)^	2020	Present the structuring of a palliative care service in order to support critical patients with COVID-19.	It is possible to structure a palliative care service quickly in crisis situations, even with little structure and in an emergency way, through cooperation, resources and teamwork, in order to offer support to patients and their families. The organized service facilitated service strategies, with goals and screening of emergency cases, prioritizing those most in need and optimizing the service. In this way, all patients and family members received adequate treatment, especially in terms of communication and contact, even in a remote format.	Qualitative descriptive study
Beyond the mask: a multidisciplinary reflection on palliating patients with COVID-19 receiving continuous positive airway pressure ventilation^(24)^	2020	Describe the experiences of professionals working in a respiratory clinical treatment unit for patients with COVID-19 in the development of guiding strategies on the cessation of respiratory ventilation when this intervention is no longer effective.	The arduous challenges that the pandemic provoked in health services, in a way, also left many lessons learned, especially on how to offer palliative care in a set of serious and emergency circumstances. Thus, it is extremely necessary that palliative care be widely discussed by a multidisciplinary team, including family members and the patient, this dynamic requires preparation, a personalized approach on a case-by-case basis, recognizing who is behind the masks and what their real needs are.	Descriptive study
Preparing a young palliative care unit for the COVID-19 pandemic in a teaching hospital in Ghana^(25)^	2020	Present strategies and suggestions to deal with the palliative care needs of critically ill patients with COVID-19 and their families, based on the experiences of a palliative care team in a hospital in Ghana-Africa.	The shared experiences in creating, quickly and urgently, a palliative care sector for patients in serious and irreversible situations of COVID-19, reveal positive and innovative strategies, such as communication and remote approximation between patients, family members and health professionals throughout the care phase and also in the grieving process. Sharing these experiences is a stimulus to other services demonstrating that it is possible to reorganize and readapt to deal with palliative care needs in emergency situations.	Qualitative exploratory study
Preparedness to Face the COVID-19 Pandemic in Hospice and Palliative Care Services in the Asia-Pacific Region: A Rapid Online Survey^(26)^	2021	Assess the readiness and capacity of palliative care services in the Asia-Pacific region to care for patients with COVID-19.	There are countless changes that occur in the lives of those who experience the consequences and mishaps of COVID-19 in the terminality of life. That is why it is essential that palliative care be quickly integrated into this assistance, this work. Recommendations are listed, important and fundamental, to be followed for the strengthening and foundation of palliative care services.	Cross-sectional study
Visitor Restrictions, Palliative Care, and Epistemic Agency: A Qualitative Study of Nurses Relational Practice During the Coronavirus Pandemic^(27)^	2021	Understand the ethical issues that palliative care nurses experience as a result of circumstances related to COVID-19 and how they deal with these issues.	Professionals from different areas of health experienced numerous negative consequences during the worsening of the pandemic. In this way, when going through these tensions, they made an effort to maintain fundamental and ethical values in their practices, engaging to integrate safety and humanity in their work, strengthening the practices advocated by palliative care, enabling an end of life with comfort, quality and bringing together (even if remote) patients and family members at the time of departure.	Qualitative descriptive study

*TX- text; COVID-19 - Coronavirus Disease 2019.*

The data analyzed in the selected collection are unanimous in reporting that health systems around the world were overwhelmed with confirmed cases of COVID-19, depleting not only the care sectors of people seeking treatment, but also physically and mentally exhausted health professionals. These conditions put the proper management of treatments at risk, causing many institutions to rethink their organizational dynamics^(15-17,19,23,25-27)^.

Although the first instinct of most people, especially health professionals, is to state that life is the most important element and, therefore, there is no reason not to try to save everyone at any cost, a premise that is questioned during the care of people with severe cases of COVID-19, most works (64%) in this review addressed the importance of understanding case progression severity and irreversibility, highlighting the need for interventions that are not curative, but rather palliative and comfort^(15-16,18,20-22,24-25,27)^.

Linked to this context, all works presented suggestions, successful experiences and palliative care strategies, which can be reproduced by other professionals, adopted during the care of patients with COVID-19 and their families, such as virtual visits addressed in 78% of selected works, restructuring of the dynamics of sectors and training of professionals to provide this care to patients, reported in 64% of studies, in addition to the interventions used to mitigate the impact of distancing between patients and family members during the hospitalization period, reported in 75% of studies.

These and other contents addressed in selected articles are detailed below. To present it in a summarized way, the findings were discussed in two themes that interact with each other: *The sudden advent of COVID-19 with impacts on palliative care;* and *The strategies used in palliative care to mitigate these impacts*.

## DISCUSSION

Since the coronavirus pandemic was decreed by the WHO, science has collaborated with numerous and important discoveries, but there is still much to discover about the disease, whether due to the periodic emergence of new variants or the appropriate clinical management of patients, especially those that progress to a serious disease condition. With the rapid progression of the pandemic and the increase in infection rates, many health services realized the need to readjust to be able to support the entire contingent of patients affected by the severe form of COVID-19^(15,23,25-26)^.

Studies have shown that the need to reorganize and adapt to this new reality was driven by the collapse experienced in services with overworked professionals, scarce resources limiting and jeopardizing care, the availability of beds and equipment, especially those with intensive use^(16,23,25-27)^. These implications were significant and, to remedy them, strong strategies were needed to ensure the well-being of patients and families as well as the safety of frontline health professionals^(16,20)^.

This overload on the health system required implementing biosafety protocols that were commonly used, but which during this period become more essential. Above all, there is a need for professionals who are more prepared to face the new coronavirus^(20)^, in addition to the adoption of restrictions on visits and stay of family members with patients which, according to most studies, was the measure that most impacted patients’ and also health professionals’ lives^(15,17,19)^.

Falling ill with COVID-19 in itself is already a negative and fearful experience, given the uncertainty of its progress, but it becomes more distressing when there is the possibility of hospitalization, where patients are separated from their families. Since the pandemic began, there have been health determinations in several countries, with a view to holding back the spread of the virus, suspending the routine of face-to-face visits and staying with patients in hospital environments^(21)^.

Visits and follow-ups during hospitalization are important and indicative of quality, aiming to maintain family ties and communication with the team. The restrictions provoked several reactions, including emotions of fear and anguish by family members when imagining that their loved one died alone as well as patients’ need for comfort when they miss their family members^(19,21,27)^.

Additionally, according to the studies, this new scenario highlighted other limitations at the clinical level in disease management, especially in cases of severe involvement, such as the need to “choose” which patients would or would not receive life-sustaining interventions^(24)^. With the increasing number of situations where severity was imminent and irreversibility was a fact, the need to increase care focused on relieving suffering and quality of life linked to conventional care became evident, with palliative care entering the scene in this context^(15,17-18,22)^.

Although palliative care is an important therapeutic modality and offers numerous benefits to patients with aggravation and poor prospects for life and their families, it still occupies a peripheral and little disseminated place in the care area, especially in the intensive specialty, a situation that according to studies^(23-24)^ is due to team unpreparedness, lack of support and institutional organization.

It is in this excess of moving and regretful scenarios, seen during the pandemic and in the face of the unpredictability and speed, with which the outcome of COVID-19 can be given, realizing the need and challenges of implementing assistance strategies in palliative care, to have the comfort and quality of life necessary to maintain patients’ dignity in a serious situation at the end of life^(18,24)^.

According to the analyzed studies, it was up to health professionals used to intensive care to recreate and readjust the way of providing care, thus promoting an interface between advanced life-saving technology and humanistic care centered on patients and their families^(15,25-27)^.

This expressive number of critically ill patients and, consequently, deaths resulting from COVID-19, brought a greater proximity to the process of death, dying and mourning in ICU, as shown in some outlines of analyzed articles, complementing that these feelings are touched on by physical distancing^(18-19)^.

Thus, according to experiences reported in studies, in order to alleviate the psychological, emotional and spiritual suffering of patients seriously affected by COVID-19, isolated in ICU without receiving visits, institutions around the world instituted visits virtual^(20-21)^. Enabling a humanistic approach in the management of these patients, this strategy made it possible for patients (those who were able to) and their families to approach virtually. Even from a distance, the studies’ conclusions report that it was possible to provide emotional support, both for patients and their families, keeping them informed about the health conditions and progress of their loved ones^(17-19,21-22)^.

In addition to allowing patients to be closer to their families, the strategy of virtual visits, telephone calls and recorded audio messages also enabled a closer relationship with the nursing team, even helping to better direct treatment, considering that many patients are submitted to mechanical ventilation, failing to express their wishes, so families act as their spokesperson^(19,21-22)^.

It is noticed among the reports observed in the descriptions of the studies that this assistance tool will become permanently present in the units, because in addition to helping in patients’ physical and psychological recovery, overcoming communication barriers with the outside world, it awakens in health professionals a deep sense of compassion. Faced with these experiences, the teams find valuable forms of reflection and confidence to face the daily mishaps imposed by the pandemic on their professional life^(15,18,21-22)^.

Although this strategy has brought countless and indisputable benefits, it still faces barriers to be overcome. The main obstacle in the practice of this strategy was time, with the increasing number of patients admitted to the ICU, and professionals who did not have the working conditions to put this task into practice^(21,25)^. Another impediment was the difficulty for the family member to have access to a device with technology to use this resource and know how to use it as well as the availability of the internet network^(21,25)^.

Another important strategy present in the studies that favored palliative care in environments such as the ICU was institutional and team planning and reorganization. The responsible allocation of human resources was fundamental for the maintenance of care and less physical overload of professionals^(16)^. However, the great demand for professionals has inevitably contributed to competition in the market and causing a shortage of specialists, which means that many hospitals face daily difficulties in meeting health teams’ needs in a number that is at least adequate for the care of critically ill patients due to COVID-19^(16,27)^.

In this context, many hospitals have organized multidisciplinary centers of expertise in palliative care in order to outline plans and strategies for the best management of critically ill patients affected by COVID-19, as well as for the general population, transmitting confidence with transparent information^(23,25-26)^.

Amidst a pandemic such as COVID-19, having a team trained in palliative care that can support the health team, brings numerous benefits not only to the professionals involved, but mainly to patients who need not only physical care, but also emotional and psychological support^(15)^. Studies also warn that, even if there is currently a decrease in the number of new cases, hospital units need to allocate resources responsibly and create more favorable conditions for healthcare professionals to act ethically, scientifically and compassionately^(15,25)^.

In addition to the fight against COVID-19, palliative care was essential, considering that it can contribute to controlling the symptoms of the disease, as well as providing psychological and spiritual support to patients and their families, helping the health team remain motivated to provide high-quality care to people who are in a state of extreme vulnerability^(18,22,27)^.

### Study limitations

It is possible to observe that most articles refer to the experiences of teams and institutions separately, which makes it difficult to elaborate a broader overview, since each one reports its particularity. Despite this reality, the findings offer positive contributions to the context of palliative care, suggesting further research that addresses this issue in a general context.

Moreover, the scarcity of work developed on the national scene was also evident, which justifies the need for research that can portray the palliative care strategies that were developed in Brazil during the pandemic.

### Contributions to nursing, health, or public policies

The works presented here certainly encourage reflections that will guide care practices in difficult times like this, the pandemic, emphasizing the need and importance of transcending technical measures for holistic interventions that address biopsychosocial aspects. These actions alleviate not only the consequences for patients, but also for their families.

Furthermore, all strategies evidenced in the studies showed positive results and represent low or no cost, i.e., are all reproducible and can be used as a model of innovative features for the implementation and reorganization of palliative care services.

## FINAL CONSIDERATIONS

Furthermore, the set of evidence pointed out in this work reinforces the numerous implications triggered by the pandemic, whether for patients with severe cases or their families, who had to face various aspects of social distancing, advocated by health agencies, and separation from their hospitalized loved one, especially in the most serious cases.

Faced with this pandemic scenario, palliative care converges into the best strategy for providing health care, used as a comfort to bring relief and comfort to patients and families. Likewise, professionals who work on the front line, by being able to apply palliative care at the end of life, reinforce the fundamental principles of respecting the life, dignity and rights of every human being in all its dimensions.
